# Evolving as a holobiont

**DOI:** 10.1371/journal.pbio.2002168

**Published:** 2017-02-28

**Authors:** Lauren A. Richardson

**Affiliations:** Public Library of Science, San Francisco, California, United States of America

Some of the most exciting recent advances in biology have been in our understanding of how the microbiome—the community of bacteria, fungi, and other single-celled microorganisms—influences host functions and behaviors. From the way we eat, to the way we think, to our susceptibility to diseases (just to name a few), the microbiome has a huge impact on human physiology. But microbiomes aren’t just for humans, or even just for mammals. The composition and function of microbiomes are critical for most animals and plants, so much so that many scientists believe that hosts and their microbiomes should be considered as single ecological unit—the holobiont. Given their ubiquity and importance, researchers are now investigating how this symbiotic relationship between hosts and microbes has evolved over time.

In a paper recently published in *PLOS Biology*, the authors investigate whether the composition of a microbiome changes in parallel with the evolution of its host species [1]. To do this, they characterized the microbiota of 24 carefully reared animal species from four different groups (*Peromyscus* deer mice, *Drosophila* flies, *Anopheles*/*Aedes*/*Culex* mosquitoes, and *Nasonia* wasps), and they also re-analyzed data from seven species of wild hominid. By comparing the composition of the microbiomes to how closely- or distantly-related these 24 species are, they determined that the more closely-related two host species are evolutionarily, the more similar their microbiota, and inversely, the more distantly-related, the more distinct (Fig 1). Thus, the microbiome isn’t a random assembly of microbes derived from the environment, but rather there has been a selection on maintaining specific host–microbiota interactions over time. The authors term this relationship between the microbiome and host evolution “phylosymbiosis”. To test this, they performed interspecific microbiome transplants in both mice and wasp species, and found that the microbiome of even closely related species was less functional than the endogenous microbiome, indicating that each host is ideally suited for its own microbiome.

**Fig 1:**
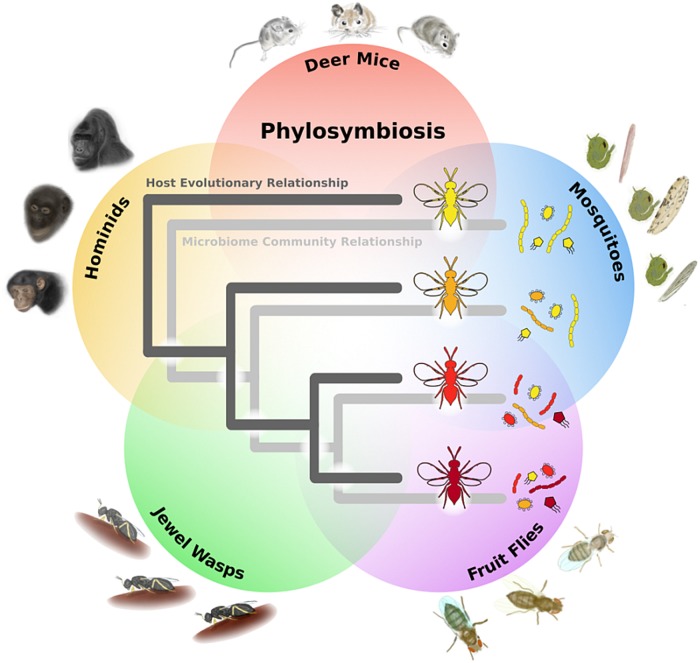
Microbiome relationships mirror host evolution. The more distantly related species are, the more distinct the composition of their cognate microbiomes, as reflected in the overlaid phylogeny of wasps and their microbiota. *Image credit*: *Andrew Brooks*, *Bordenstein Lab*.

To further understand the process of phylosymbiosis, researchers can mimic it in the lab via artificial selection. In work published in the journal *Frontiers in Microbiology*, researchers performed a 15-generation selection experiment on bank voles (*Myodes glareolus*) and found that voles bred for the ability to maintain body mass on a high-fiber, herbivorous diet had a distinct microbiome compared to control lines [2]. Similarly, chickens bred for either high weight or low weight (while maintaining the same diet and husbandry) have altered gut microbiota; in a *PLOS ONE* study, researchers characterized the composition of the microbiome of chickens that had been bred for >50 generations for these specific phenotypes [3]. They identified families of bacteria that were moderately heritable, and found that some interactions among gut microbiota members are mediated by the host genes. These studies demonstrate that the make-up of the microbiome is not dictated solely by diet and environment, but that rather it changes in conjunction with the demands of the host.

One important technique needed to understand holobionts is a computational framework for modeling host-microbiota interactions. Published in *PLOS Computational Biology*, researchers present one such model, called a neutral model [4]. This model assumes that microbes have no effect on the fitness, or reproductive success, of the host, which simplifies the number of assumptions needed to generate the model. Despite this simplification, the model provides insights into how microbiomes are acquired and assembled from the environment, and will allow for future research on the more complex nature of host-microbe interactions.

Another method to investigate the path of evolution is to compare multiple modern species. To parse the contribution of host genetics from that of the environment on the gut microbiome of nematode worms, an article from *Frontiers in Microbiology* catalogued the microbiomes of several members of the *Caenorhabditis* genus spanning 200–300 million years of evolution [5]. The worms were raised in various soil environments, and both the microbiome and the environmental bacterial populations were sequenced. The scientists found that environment did have a large impact, but there was also a significant contribution of host genetics. Interestingly, while their sequencing data were not able to distinguish specific host-associated microbial taxa, the authors demonstrated that particular species of *Enterobacteriaceae* could protect their own host from infection by *Pseudomonas aeruginosa* but not a non-host, indicating that this protection is mediated by an evolved host-microbe interaction.

In addition to comparing different species, researchers can study how the genetic variation within a species influences the microbiome. In a *Genome Biology* article, the authors compared microbiome composition across 15 body sites to the genetic variation within 93 human individuals [6]. This study found that differences in the microbiome are associated with host genetic variants related to genes involved in the immune response, and these variants are strongly associated with microbiome-related disorders like inflammatory bowel disease. Intriguingly, these genomic regions differ substantially between human populations, suggesting that there has been recent adaptation to environment-specific microbiomes.

As mentioned above, many aspects of plant physiology are also dependent on the microbiome. In another article published in *PLOS Biology*, scientists investigated how abiotic and host factors contribute to microbiome community structure on the surface of the model plant *Arabidopsis thaliana* [7]. Interestingly, they found that within the community, there is a complex network of microbe-microbe interactions that also influence community structure on the leaf. The authors identify specific microbes—which they term “hub microbes”—that are critical for determining the make-up of the community. These hub microbes included fungi and oomycetes, in addition to bacteria, and can alter the growth and diversity of community members. Both abiotic and host genetics can impact these hub microbes, which then transmit the effect to the microbial community. This work highlights the complexity of factors that can alter holobiont structure and proposes that hub microbes are potential targets for engineering microbial communities and for future biocontrol.

One constituent of the microbiome that scientists hope to target is the methane-producing archaea found in the gut of ruminants (e.g. cattle, goats, sheep). Methane is a potent greenhouse gas, and ruminants are the major source of methane emissions. By comparing the methane emissions and archaeal abundance from related groups of cattle, the authors of a *PLOS Genetics* article show that archaeal abundance is under host genetic control [8]. Thus, metagenomic profiling of methanogenesis genes can be used as a criterion to select animals for breeding programs designed to reduce methane production. In this way, scientists will be taking advantage of holobiont variation to develop a steak with a smaller impact on climate change.

For more detailed reading please see the associated PLOS Collection [9].

## References

[1] Brooks AW, Kohl KD, Brucker RM, van Opstal EJ, Bordenstein SR. Phylosymbiosis: Relationships and Functional Effects of Microbial Communities across Host Evolutionary History. PLOS Biol. 2016; 14(11): e2000225 10.1371/journal.pbio.2000225 27861590 PMC5115861

[2] Kohl KD, Sadowska ET, Rudolf AM, Dearing MD, Koteja P. Experimental Evolution on a Wild Mammal Species Results in Modifications of Gut Microbial Communities. Front Microbiol. 2016; 7:634 10.3389/fmicb.2016.00634 27199960 PMC4854874

[3] Meng H, Zhang Y, Zhao L, Zhao W, He C, Honaker CF, et al Body Weight Selection Affects Quantitative Genetic Correlated Responses in Gut Microbiota. PLOS ONE. 2014; 9(3): e89862 10.1371/journal.pone.0089862 24608294 PMC3946484

[4] Zeng Q, Sukumaran J, Wu S, Rodrigo A. Neutral Models of Microbiome Evolution. PLoS Comput Biol. 2015; 11(7): e1004365 10.1371/journal.pcbi.1004365 26200800 PMC4511668

[5] Berg M, Zhou XY, Shapira M. Host-Specific Functional Significance of Caenorhabditis Gut Commensals. Front Microbiol. 2016; 7:1622 10.3389/fmicb.2016.01622 27799924 PMC5066524

[6] Blekhman R, Goodrich JK, Huang K, Sun Q, Bukowski R, Bell JT, et al Host genetic variation impacts microbiome composition across human body sites. Genome Biol. 2015; 16:191 10.1186/s13059-015-0759-1 26374288 PMC4570153

[7] Agler MT, Ruhe J, Kroll S, Morhenn C, Kim ST, Weigel D, et al Microbial Hub Taxa Link Host and Abiotic Factors to Plant Microbiome Variation. PLOS Biol. 2016; 14(1): e1002352 10.1371/journal.pbio.1002352 26788878 PMC4720289

[8] Roehe R, Dewhurst RJ, Duthie CA, Rooke JA, McKain N, Ross DW, et al Bovine Host Genetic Variation Influences Rumen Microbial Methane Production with Best Selection Criterion for Low Methane Emitting and Efficiently Feed Converting Hosts Based on Metagenomic Gene Abundance. PLOS Genet. 2016; 12(2): e1005846 10.1371/journal.pgen.1005846 26891056 PMC4758630

[9] In PLOS Collections. http://collections.plos.org/open-highlights-holobiont

